# Molecular Immune Mechanism of Intestinal Microbiota and Their Metabolites in the Occurrence and Development of Liver Cancer

**DOI:** 10.3389/fcell.2021.702414

**Published:** 2021-12-08

**Authors:** Chenchen Bi, Geqiong Xiao, Chunyan Liu, Junwei Yan, Jiaqi Chen, Wenzhang Si, Jian Zhang, Zheng Liu

**Affiliations:** ^1^ Department of Pharmacology, Medical College of Shaoxing University, Shaoxing, China; ^2^ Department of Oncology, Affiliated Hospital of Shaoxing University, Shaoxing, China; ^3^ Department of Clinical Medicine, Shaoxing People’s Hospital, Shaoxing, China; ^4^ Department of General Surgery, Affiliated Hospital of Shaoxing University, Shaoxing, China

**Keywords:** intestinal flora, metabolites, liver cancer, occurrence and development, immune mechanism, therapeutics

## Abstract

Intestinal microorganisms are closely associated with immunity, metabolism, and inflammation, and play an important role in health and diseases such as inflammatory bowel disease, diabetes, cardiovascular disease, Parkinson’s disease, and cancer. Liver cancer is one of the most fatal cancers in humans. Most of liver cancers are slowly transformed from viral hepatitis, alcoholic liver disease, and non-alcoholic fatty liver disease. However, the relationship between intestinal microbiota and their metabolites, including short-chain fatty acids, bile acids, indoles, and ethanol, and liver cancer remains unclear. Here, we summarize the molecular immune mechanism of intestinal microbiota and their metabolites in the occurrence and development of liver cancer and reveal the important role of the microbiota-gut-liver axis in liver cancer. In addition, we describe how the intestinal flora can be balanced by antibiotics, probiotics, postbiotics, and fecal bacteria transplantation to improve the treatment of liver cancer. This review describes the immunomolecular mechanism of intestinal microbiota and their metabolites in the occurrence and development of hepatic cancer and provides theoretical evidence support for future clinical practice.

## Introduction

Liver disease is a critical and common disease, which represents a main health burden worldwide, with increasing morbidity and mortality (Acharya and Balaji., 2021). Globally, liver cirrhosis and liver cancer are the 11th and 16th most common causes of death, respectively (Asrani et al., 2019). Chronic liver disease (CLD) is caused by viral hepatitis, nonalcoholic fatty liver disease (NAFLD) and alcoholic liver disease (ALD), and can result in hepatocellular carcinoma (HCC) (Avila et al., 2020; Schwabe et al., 2020; Schwabe and Greten, 2020). Therefore, an active treatment for CLD is urgently required, especially liver cancer. However, there are currently no effective treatment measures for HCC because of its complex pathophysiological mechanism.

Recent studies have illustrated the association between intestinal microbiota and carcinogenesis, which is significant for the pathogenesis of liver cancer (McQuade et al., 2019; Kanmani et al., 2020; Schwabe and Greten, 2020). Increasing evidence has demonstrated the changes in gut microbiota composition and function play a critical role in liver health from pre-cirrhotic stages to cirrhosis, decompensation, and the requirement of liver transplantation (Schwabe et al., 2020). The microbiota-gut-liver axis is the interaction between the liver, gut, and intestinal microbiota (Tripathi et al., 2018a). Intestinal-derived metabolites, cellular components, hormones, and other substances are transported into the liver through the portal circulation and interact with immune cells, causing inflammatory reactions and inducing the progression of various hepatic diseases. The liver is directly related to the gastrointestinal tract through the portal venous circulation and biliary system, so it is often exposed to bacterial products produced by intestinal microorganisms. The pathogenesis of HCC is linked to negative alterations to the gut microbiota, and the liver is connected to the intestine directly through the hepatic portal circulation (Gupta et al., 2019). However, the molecular mechanism underlying the role of the gut microbiota in hepatocarcinogenesis remains unclear.

Numerous studies have demonstrated that the association between the gut microbiota and the immune system may be a critical cause of hepatocarcinogenesis. The microbe-gut-liver axis plays an important role in liver cancer (Albillos et al., 2020). Various factors, such as drinking alcohol, high fat-diet, and bacterial infection can perturb the intestinal flora and destroy the intestinal barrier. This disruption results in the release of intestinal toxins and metabolites that target the liver and trigger the liver immune response *via* Kupffer cells (KCs), macrophage and neutrophil activation, and cell surface receptors combined with bacterial pathogen-associated molecular patterns (PAMPs); together, these processes activate a series of reactions that can lead to liver damage (Golonka and Vijay-Kumar., 2021). The liver may eventually develop hepatitis, cirrhosis, fibrosis, and even cancer. Intestinal bacteria and their metabolites also promote the occurrence and development of liver cancer through their receptors or inflammatory signal passageway. For example, intestinal bacteria ferment butyric acid, which is produced by non-absorbable fiber inulin, and can promote liver cancer (Wisniewski et al., 2019). Moreover, under the function of intestinal bacteria, primary bile acids can generate secondary bile acids, which will promote the occurrence and development of liver cancer (JM et al., 2014). Thus, intestinal bacteria and their metabolites are essential in the development of liver cancer and have attracted wide attention.

The gut microbiota affects immune responses, and studies have revealed a strong association between the gut microbiota and the response to immune checkpoint blockade. Therefore, methods of regulating the microbiome are being developed for various cancers. These methods include the use of fecal microbiota transplant, probiotic administration, and dietary intervention, all of which are being used experimentally for the treatment of liver cancer (McQuade et al., 2019).

Here, we summarize the molecular immune mechanism of gut microbiota in the occurrence of liver cancer and reveal the important role of the microbiota-gut-liver axis. In addition, we describe how the intestinal flora can be balanced by antibiotics, prebiotics, postbiotics, and fecal bacteria transplantation to improve the treatment of liver cancer. In conclusion, we summarize the immunomolecular mechanism of gut microorganisms and their metabolites in the occurrence of liver cancer, and provide theoretical evidence and support for future clinical practice.

## Liver Diseases and the Gut Microbiome

The gut and liver are interconnected and interact with each other. Anatomically, the gut microbiota and their metabolites, enterogenous hormones, and nutrients help to maintain liver function and metabolism, while the liver absorbs these products from the gut and secrets bile acids to the gut (Delacroix et al., 1982). Disruptions of this interaction may lead to the development of liver diseases such as liver inflammation, ALD, NAFLD, non-alcoholic steatohepatitis (NASH), fibrosis, and cirrhosis (Seki et al., 2007; Cassard and Ciocan., 2018).

### ALD

In addition to the direct toxicity of alcohol on hepatocytes cells, the pathogenesis of ALD is also related to gut microbiota disorder, loss of intestinal barrier function and activation of Toll-like receptors (TLRs) on hepatic immune cells (Albillos et al., 2020). Alcohol intake influences the gut microbiome, which occurs long before fibrosis develops (Bull-Otterson et al., 2013). In chronic alcoholism patients with jejunal inhalation, the intestinal overproduction of aerobic and anaerobic microorganisms has been widely recognized. In mice fed alcohol or in individuals with chronic ethanol abuse, metagenomic analysis of the intestinal microbiome has shown that bacterial diversity decreased and phylogeny transited to higher protein bacterial abundance (Chen et al., 2011; Yan et al., 2011; Mutlu et al., 2012). Interestingly, a specific microbial pattern, including a large number of *Bifidobacteria* and *Streptococci*, has recently found in the intestines of patients with severe alcoholic hepatitis (Llopis et al., 2016).

### NAFLD

NAFLD can be broadly divided into non-progressive phenotype liver diseases, NAFL and NASH. NAFLD, closely related to obesity, has common mechanisms with type 2 diabetes mellitus, insulin resistance, and risk factors of cardiovascular disease. Previous studies have shown that a high-fat diet can change the microbiota, thereby damaging the intestinal barrier (Mouries et al., 2019), promoting the portal influx of bacterial products, aggravating non-liver inflammation, and generating metabolic abnormalities. Although a causal link between NAFLD and gut microbiota remains unclear, numerous studies have emphasized the effect of the gut microbiome in the pathogenesis of NAFLD. Indeed, preclinical studies have revealed that the prevalence of intestinal bacterial overgrowth (Kapil et al., 2016; Miele et al., 2009) and the changes in microbiota composition in NAFLD patients (Boursier et al., 2016) were higher than those in healthy controls. Using shotgun metagenomics sequencing, researchers have found a correlation between the microbiological characteristics of patients with NAFLD and advanced fibrosis, which is characterized by the increased abundance of *Vulvobacterin* and *Escherichia coli* (Ren et al., 2019). Although NAFLD and ALD are the basic mechanisms of intestinal barrier dysfunction, there are subtle differences in their intestinal microbial composition, intestinal permeability, bile acids, and ethanol and choline metabolites, which need to be further studied.

### Viral Hepatitis

Viral hepatitis, mainly including hepatitis A virus (HBV), hepatitis B virus (HBV), hepatitis C virus (HCV), hepatitis D virus (HDV), and hepatitis E virus (HEV), is an infectious disease that seriously harms human health. Several studies have shown that gut dysbiosis is associated with viral hepatitis, chiefly HCV and HBV infection. HCV infection is a prime cause of cirrhosis, HCC, liver failure, and death (Craxì et al., 2008). Liver failure and disease progression in patients with HBV infection has been found to be linked to gut flora dysbiosis. HBV infection delays the development of the gut microbiota, and alters the dynamic changes in gut microbiota. One study found that the gut bacterial distribution in HBV infected mice was dominated by six phyla, namely, Bacteroidetes, Firmicutes, Verrucomicrobia, Proteobacteria, Actinobacteria, and Spirochaetes (Wang et al., 2019). Similarly, dysbiosis of intestinal flora can affect the progression of viral hepatitis.

### Cholestatic Hepatitis

Cholestatic hepatitis is a type of bile duct excretion dysfunction with multiple causal factors. In patients with cholestatic hepatitis, bile cannot be actively discharged through the bile duct to the intestine and instead refluxes into the blood. One study identified seven genera, with different abundances between the intrahepatic cholestatic of pregnancy and healthy individuals. The seven genera included Escherichia/Shigella, Parabacteroides, Flavonifractor, Atopobium, Turicibacter, *Lactobacillus*, and Megamonas. In addition, the family Lactobacillaceae and the phylum Proteobacteria were also different between the two groups (Molinaro et al., 2018). Another study suggested that intestinal barrier function can be affected by cholestasis in the intestinal lumen (Abu Faddan et al., 2017). Thus, breaking the balance between the bile acids and gut microbiota can lead to inflammation.

### Cirrhosis

Liver cirrhosis is accompanied by severe intestinal barrier impairment. The damage of the intestinal barrier in patients with decompensated cirrhosis is due to the damage of all levels of intestinal barrier defense, and is related to liver dysfunction, reduction of bile flow, and immune function impairment. Intestinal barrier dysfunction and the gut microbiome directly participate in the pathogenesis of compensated cirrhosis, and both are related to the incidence and severity of complications of decompensated cirrhosis, namely bacterial infection and encephalopathy. For decades, human and experimental liver cirrhosis models have highlighted changes in intestinal microbiota and bacterial overgrowth (Shah et al., 2017).

Currently, metagenomic techniques have been used to characterize the fecal microbiome in cirrhosis as one of reduced diversity, increased relative overgrowth of potentially pathogenic bacterium, such as Enterobacteriaceae*,* Staphylococcaceae and Enterococcaceae, and decreased relative abundance of the potentially beneficial bacterium Lachnospiraceae and Ruminococcaceae (Bajaj et al., 2013; Chen et al., 2011; Qin et al., 2014). The change in microbial composition in cirrhosis is the result of microbiome management: the decrease in intestinal movement and transport time occurs mainly at the ascites stage (Pérez Paramo et al., 2000; Gunnarsdottir et al., 2003; Yan et al., 2011); bile acid abnormalities, including a decrease in primary bile acids (PBAs) level and an increase in intestinal secondary bile acids (SBAs) level (Lorenzo-Zuniga et al., 2003; Kakiyama et al., 2013, 2014); and impaired intestinal immune function. Experimental cirrhotic ascites is related to impaired Paneth cell alpha-defensins and damage to dendritic cells (DCs) (Teltschik et al., 2012), both of which are particularly severe in rats with ascites and pathological bacterial translocation. Another factor leading to microbiota changes is hypochloremia during cirrhosis (Llorente et al., 2017). Remarkably the abnormal pattern of pathogenic bacteria is an independent factor in liver cirrhosis (Chen et al., 2011; Kakiyama et al., 2013).

## Role of the Microbe-Gut-Liver Axis in the Occurrence and Development of Liver Cancer

Recently, increasing evidence has shown that the destruction of the gut-liver axis results in the progression of most CLDs, including cirrhosis and liver cancer (Kanmani et al., 2020). Dysbiosis and leaky gut are extraordinary characteristics of liver cirrhosis, which result in an increase in intestinal bacterial infections such as spontaneous bacterial peritonitis, which are considered to be responsible for the development of liver cancer in patients with cirrhosis. Moreover, striking features are observed at all phases of CLD, accelerating the gradual progression from fibrosis to cirrhosis and HCC (Tripathi et al., 2018b; Yu and Schwable., 2017). Moreover, intestinal leakage and intestinal disorders are linked; intestine leakages make bacterial metabolites and microbe-associated molecular patterns (MAMPs) easier to transpose and reach the liver, while intestinal disorders can lead to the formation of more permeable intestinal barriers (Schwabe and Greten, 2020).

### Gut Leakage

The cirrhotic liver serves as the initial site for detoxification of microbial products from portal blood. “Leaky gut” refers to a situation of increased gut permeability for microbiota and their metabolites, which often occurs in hepatic cirrhosis and represents an important pathogenetic factor for major complications. Prolonged gut transit induces intestinal bacteria overgrowth, a pathological condition in which colon bacteria translocate into the small intestine. Furthermore, intestinal flora disorders have been detected in patients with cirrhosis of the liver, characterized by excessive growth of potentially pathogenic bacteria and a decrease in non-pathogenic native bacteria (Henao-Mejia et al., 2012). Using high-molecular weight polyethylene glycol or FITC-dextran methods, intestinal leakage can be examined in patients or animal models of ethanol-associated and biliary liver disease (Parlesak et al., 2000; Yan et al., 2011; Fouts et al., 2012).

The mechanism underlying leaky gut is multifactorial and has been studied previously (Pradere et al., 2010; Yu and Schwabe, 2017). LPS, a component of the cell wall of Gram-negative bacteria that induces an inflammatory reaction *via* TLR4, is the most commonly used marker for inflammatory bacterial translocations. The primary levels of LPS gradually increase throughout the course of CLD, with the highest levels observed in patients with stage C cirrhosis of Child Pugh (Lin et al., 1995). Similarly, bacterial DNA levels of TLR9 agonists were elevated in CLD (Bellot et al., 2010). These results indicate that livers with chronic lesions are likely to be exposed to a wide range of TLR ligands as well as other bacterial metabolites. Moreover, an increase in ethanol metabolism in the gut due to excessive alcohol consumption, promotes intestinal dysfunction and excessive bacterial growth, resulting in intestinal leakage. This results in liver damage, which facilitates the mitosis of liver cells and inhibits apoptosis, ultimately leading to the development of HCC (Méndez-Sánchez et al., 2020).

### Dysbiosis

When stressed by various disease processes, the human gut microbiome experiences dysbiosis, which promotes the progression of liver fibrosis/cirrhosis by increasing the inflammatory reaction and progressing toward fibrosis/cirrhosis (Albillos et al., 2020). For instance, alcohol consumption induces direct hepatoxicity by microbiota dysbiosis, which results from bacterial proliferation in the small/large intestine or direct microbial toxicity, and by immediate local lesion of the intestinal barrier, leading to increased bacterial translocation and inflammation (Mutlu et al., 2012; Leclercq et al., 2014; Meroni et al., 2019).

Recently, studies have shown the occurrence of dysbiosis at various stages of CLD. The most relevant studies reveal gut dysbiosis in cirrhosis (Qin et al., 2014; Pasolli et al., 2016; Ponziani et al., 2019; Ren et al., 2019), mainly during the progression stage of liver cancer and those in patients with liver cancer (Grąt et al., 2016; Ponziani et al., 2019; Ren et al., 2019) Clinical studies have demonstrated that the presence of liver cirrhosis can be accurately predicted by the change in the intestinal microflora (Pasolli et al., 2016), and emphasized that end-stage CLD is the most associated-dysbiosis disease. Dysbiosis alters various processes that influence the development of CLD and the subsequent progression of liver cancer, including inflammation, lesions, fibration and regeneration. Although the most significant changes in the gut microbiota have been observed between healthy controls and patients with liver cirrhosis (Qin et al., 2014; Pasolli et al., 2016), there is growing evidence of differences between patients with liver cirrhosis and those with HCC (Grat et al., 2016; Ponziani et al., 2019; Ren et al., 2019). Indeed, in patients with HCC and cirrhosis, excessive growth of *E. coli* in the gut was first reported in 2016 (Grat et al., 2016).

**FIGURE 1:**
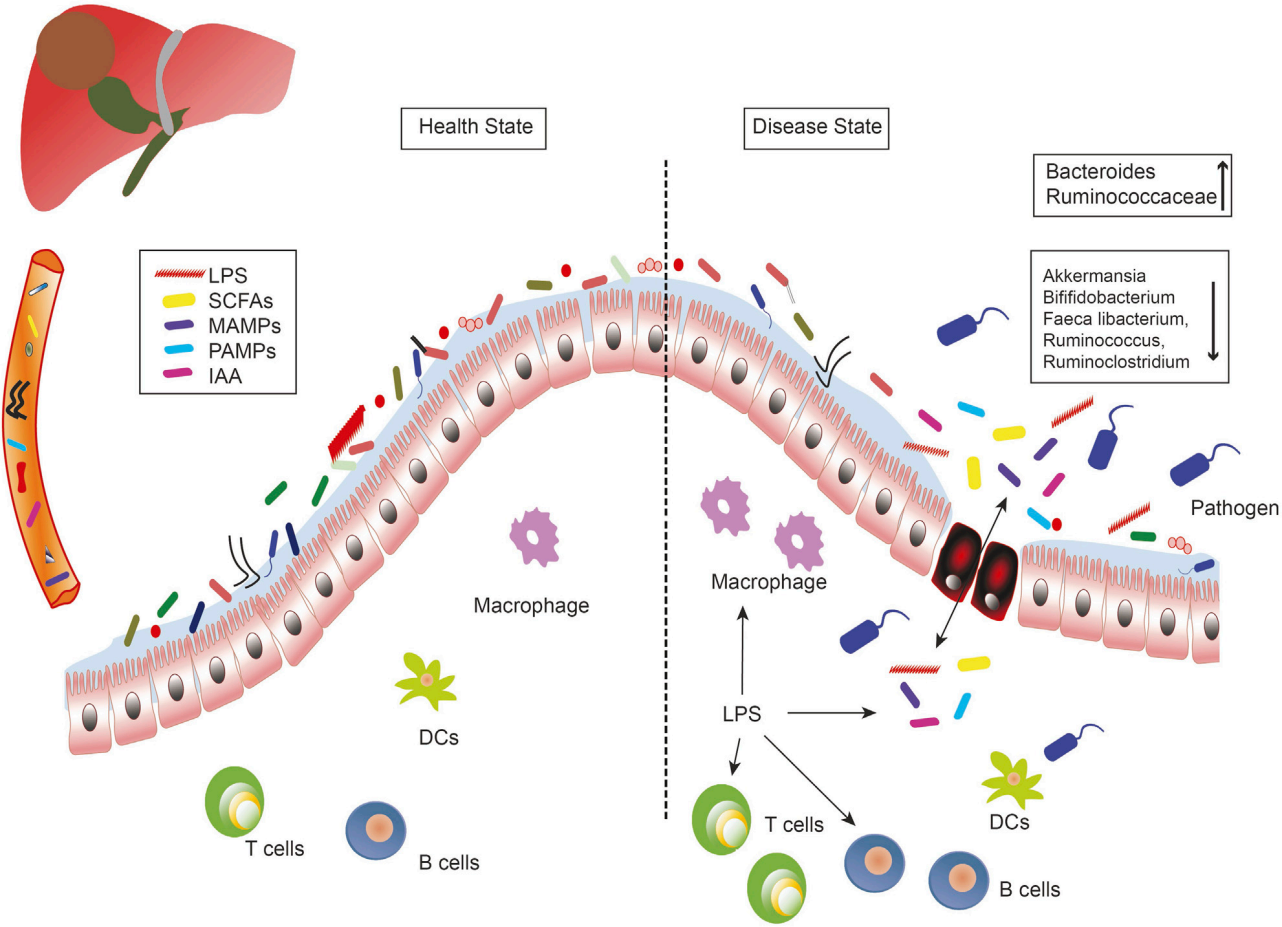
The role of the microbe-gut-liver axis in the occurrence and development of liver cancer. Intestinal microorganisms, metabolites (SCFAs, IAA), and bacterial components (LPS, MAMPs, PAMPs) are transported to the liver through the portal vein, where they interact with immune cells and generate inflammatory responses to induce liver diseases. Intestinal leakage and dysregulation of flora are the important characteristics of the liver disease. DCs, dendritic cells; IAA, indole acetic acid; LPS, lipopolysaccharides; MAMPs, microbe associated molecular patterns; PAMPs, pathogen associated molecular patterns; SCFAs, short-chain fatty acids.

The microbiome study showed a decrease in fecal bacterial diversity from healthy controls to patients with liver cirrhosis, but an increase from patients with cirrhosis to those with early liver cancer with cirrhosis. Compared to NASH-induced cirrhosis without HCC, the abundance of Ruminococcaceae and Bacteroides increased, while the abundance of Akkermansia and Bifidobacterium decreased in patients with HCC (Ponziani et al., 2019). Moreover, researchers observed that in patients with HBV-related HCC, the richness in fecal microbiota was remarkably greater than in healthy groups and those with non-HBV non-HCV (NBNC)-related HCC. Furthermore, the feces of patients with NBNC-related HCC harbored more potential pro-inflammatory bacteria including Escherichia-Shigella and Enterococcus, and a decreased abundance of Faeca libacterium, Ruminococcus, and Ruminoclostridium, contributing to the reduction of anti-inflammatory short-chain fatty acids (SCFAs) (Liu et al., 2019). (Figure 1)

Variations in the microbial profile of liver cancer have been observed in previous studies, which may be due to differences in etiology, geographic area, and nutrient intake. In addition, the differences between patients with cirrhosis and HCC and those with cirrhosis of the liver are less marked than those observed between healthy patients and those with liver cirrhosis. Therefore, not only is the microbiome-based diagnostic test more potent than the liver cirrhosis screening test for liver cancer, it is also possible that microorganisms that have a functional impact on the development of liver cancer are primarily affected by cirrhotic alterations rather than specific HCC alterations.

## Role of Gut Microbiota and Their Metabolites on the Immune Mechanisms of the Progression of Liver Cancer

### LPS and TLRs

Intestinal microorganisms can affect the health of their hosts by producing cellular components such as LPS, peptidoglycans, lipoteichoic acid, flagellin, and DNA. The cellular components are transmitted from the portal circulation to the liver, where they actively interact with immune cells, causing inflammation and the progression of various liver diseases. Among the bacterial components, LPS is a key inflammatory molecule, which is increasingly translocated to the liver during intestinal dysbiosis (Kanmani et al., 2020). LPS can induce a systemic proinflammatory and fibrotic condition, where the transduction of insulin signals is disrupted, leading to an increase in the net lipidization of adipose tissue and the transport of free fatty acids from adipose tissue to the liver (Kang et al., 2017). Once excessive lipids are exposed to cellular stress in the liver, they are ultimately expanded through the proinflammatory environment of the liver and system. Subsequently, an acute reaction and liver fibrosis develop, which ultimately progress to cirrhosis of the liver (Kumar et al., 2017), and even liver cancer.

PAMPs and metabolites are derived from the actions of the intestinal microbiome on exogenous (food and environmental exposure) and endogenous substrates (amino acids and bile acids), which can reach the liver through the portal route and promote inflammatory reactions. In 1989, Charles Janeway first proposed that pattern recognition receptors (PRRs) recognize PAMPs to activate innate immunity and adaptive immunity (Janeway, 1989). TLRs, type-I transmembrane proteins, were the first identified PRRs to be capable of sensing pathogen infection. TLRs are the main receptors in cirrhosis for the identification of gut bacteria and represent an essential component of the congenital immune system, triggering the cascade of signals associated with the progression of cirrhosis. Among the ten human TLRs, TLR2, TLR4, TLR5, and TLR9 identify bacterial infection. TLR4 is the most widely studied PRR. Through LPS-binding protein, CD14 and myeloid differentiation protein 2 (MDP2), TLR4 can recognize and bind to LPS to produce response (Wen et al., 2017). Preclinical studies have confirmed that the contribution of PAMPs to NAFLD liver damage by a reduction in liver degeneration, inflammation, and fibrosis in mice with TLR4-or TLR9 deficiencies with a high-fat or low-choline diet (Rivera et al., 2007; Saberi et al., 2009; Miura et al., 2010). In addition, the absence of inflammatory microorganisms associated with changes in intestinal flora in mice led to steatosis and inflammation of the liver through the endovascular flow of TLR4 and TLR9 agonists, ultimately resulting in increased expression of TNF-αand inflammation in the liver, which is particularly severe in mice with liver steatosis (Henao-Mejia et al., 2012). Unlike TLR4 identifying Gram-negative bacteria, TLR2 primarily identifies Gram-positive bacteria. Lipoteichoic acid is a bacterial component of the intestine which, as a ligand of TLR2, contributes to the development of HCC in obese mice by increasing tumor-promoting senescence-associated secretory phenotype (SASP) of hepatic stellate cells (HSCs) and COX2 expression. COX2-induced prostaglandin E2 (PGE2) neutralizes anti-tumor immunity and facilitates the progression of HCC. TLR5 and TLR9 identify Gram-positive and Gram-negative bacterial flagellin and CpG DNA, respectively.

### Immune Cells in Liver Cancer

#### Monocytes/Macrophages

Under the stimulation of endotoxin, monocytes from circulating sources can infiltrate the liver and differentiate into macrophages, which promote inflammatory mediators and oxygen free radicals to induce liver dysfunction and even liver failure. Monocyte-derived macrophage transformations are key events in hepatic inflammation (Zhao et al., 2018). In the liver, KCs and bone marrow-derived macrophages identify PAMPs from enterohepatic circulation *via* TLR4. TLR4 upregulation facilitates binding with its ligand, myeloid differentiation primary reaction 88, and leads to activation of mitogen-activated protein kinase (MAPK) and c-Jun N-terminal kinase (JNK), and subsequent activation of the NF-κβ pathway. This in sequence induces the release of TNF-α, IFN-γ, prostaglandin-2, IL-1α, IL-1β, IL-6, reactive oxygen species (ROS), and nitric oxide, to maintain the liver inflammatory reaction. NF-κB also activates anti-apoptotic genes with important carcinogenic effects. An increase in TNF-α production has been shown to decrease tight junction (TJ) proteins and leads to intestinal barrier damage (Méndez-Sánchez et al., 2020). In addition, the activation of the TLR4 signaling pathway and intestinal bacteria accelerates the progression of liver cancer through mediating increased cell proliferation, expression of hepatomitogen epiregulin, and inhibition of apoptosis (Dapito et al., 2012).

KCs are resident macrophages in the liver and are essential to maintain immune tolerance by phagocytosing toxins from bacteria and xenobiotics. In the case of KC depletion, circulating monocytes can adopt the transcriptional profile of KCs. In the KC niche, interactions with HSCs, hepatocytes, and endothelial cells can also trigger monocyte recruitment. A cascade of interactive signals from Notch, liver X receptor-α, and transforming growth factor-β1 (TGF-β1) recruits monocytes to fill the population with liver macrophages into KCs. Similar to cyclic monocytes/macrophages, KCs can differentiate into M1-and M2-type cells in the chronic inflammatory process of the liver, but these subtypes serve as a double-edged sword in the context liver health. For NAFLD and NASH, M2-polarized macrophages play a protective role in resisting apoptosis and avoiding lipid accumulation, while a predominance of M1 macrophages aggravates these liver diseases. In contrast, *in vitro* studies of mature HCC cell lines and clinical HCC specimens (such as blood and liver tissue) have shown that a predominant M2 phenotype in liver cancer promotes tumor growth, cell migration, metastasis dependent on epithelial-mesenchymal transformation, drug resistance, and facilitates an immunosuppressive tumor microenvironment. In addition, intestinal flora disorder induces the production of IL-25 and promotes the progression of liver cancer by activating macrophage substitution and CXCL-10 secretion in the tumor microenvironment (Li et al., 2019).

#### Neutrophils

The neutrophil/lymphocyte ratio has been widely defined as a biological marker for diagnosis, prognosis, overall survival, preoperative, postoperative, and relapse of HCC. The spectrum of cytokines surrounding the tumor microenvironment can determine whether neutrophils differentiate as N1 or N2 immunosuppressant anti-tumor subtypes. In the early-stage of liver cancer, liver cancer-associated neutrophils (LCANs) are located in the periphery and have a cytotoxic N1 phenotype for tumor cells. Neutrophils differentiate into N2 phenotypes at an advanced stage of liver cancer, due to a shift in cytokine production in favor of CCL2 and CCL17, as well as an up-regulation of TGF-β. Thus, neutrophil and TGF-β blockage can slow the growth of liver cancer, and, in particular, inhibition of TGF-β can transform the LCAN population from an N2 to N1 phenotype. The intestinal microflora has no direct effect on the pathology of LCAN in HCC. However, the diversity and composition of intestinal microorganisms can affect the circulation and the level of liver neutrophils. Thus, the greater the diversity of intestinal flora, the lower the proportion of neutrophils/lymphocytes in the blood (Golonka and Vijay-Kumar., 2021).

#### Lymphocytes

When viable bacteria cross the barrier from the intestinal lumen, bacteria are commonly translocated from the gut to the hepatic or circulatory system. KCs activate the congenital activate immune response by releasing cytokines and producing ROS. Released cytokines stir up immune cells such as neutrophils and monocytes in the liver to control invasive microorganisms, but they also promote liver damage. The supplement of lymphocytes and neutrophils and the activation of HSCs occur, which ultimately leads to collagen production and fibrosis. Finally, constant natural killer cells (iNKT), usually present in the intestine, can also migrate into the liver during bacterial translocations caused by chronic alcohol abuse, and have been found to contribute to the apoptosis of liver cells (Bruellman and Llorente., 2021). In addition, the reduction of the intestinal microflora through antibiotic treatment inhibits the development of HCC in mice by increasing the accumulation of NKT cells in the liver and CD4^+^ or CD8^+^ memory T cells (Ma et al., 2018). Higher levels of PBAs, CXCL16, and CXCR6 can alter the accumulation of NKT cells in the liver cancer mice.

#### DCs

DCs in the gut can carry bacteria to mesenteric lymph nodes, causing a more localized immune response. Prolonged inflammation can impair the functioning of the lymphatic system and further damage the immune system of the liver. One of the main mechanisms of protection of the liver for the host is the secretion of IgA in the bile attached to bacteria. Disturbances in the host can cause the liver’s immune system to further regulate the microflora to protect against damage to the intestinal barrier. More recently, DCs have received increasing attention in the cellular treatment of tumors (Montico et al., 2017; Saxena and Bhardwaj., 2017). Immature dendritic cells (imDCs) have the ability to phagocytize and process the antigen, but their ability to present antigen is low. In both *in vivo* and *in vitro* studies, various methods have been used to induce imDCs maturation to increase their antitumor activity (Crottes et al., 2016). Researchers have used different antigenic formulas to modify and charge DCs before injecting them into animal models or patients, resulting in anticancer effects (Funda et al., 2018). (Figure 3).

### Gut Microbiota Metabolites and Liver Cancer

#### PBAs and SBAs

In the liver, cholesterol is metabolized into PBAs, which are stored in and released from the gallbladder into the small intestine, and they can be used to dissolve lipids and fat-soluble dietary vitamins (JM et al., 2014). Considerable amounts of PBAs are reabsorbed from the ileum into the liver; a small proportion (∼3%) are easily deconjugated, which enters the large intestine and metabolized into SBAs by gut bacteria (Jia et al., 2018). SBAs are increasingly considered as cancer-promoting metabolites. The intestinal microflora has been shown to use bile acids as messengers to impair immune functions and influence anti-cancer effects (Wahlstrom et al., 2016; Ma et al., 2018). Studies have suggested that Gram-positive bacteria will accumulate in mice fed a high-fat diet with enhanced bile acid processing capacity (Yoshimoto et al., 2013). Thus, when the antibiotic vancomycin inhibits endogenous production of deoxycholic acid (DCA), a high-lipid diet leads to an increase in the levels of DCA produced by bacteria, together with dimethylbenzanthracene and high-fat diet, which contributes to the development of liver cancer (Yoshimoto et al., 2013).

The liver is sensitive to the metabolites of intestinal bacteria and changes in the gut microflora affect the functioning of the liver’s immune cells. The intestinal microorganisms participate in the physiological activity of the host by acting on the pool of bile acids, and thus regulate hormonal secretion and immunization by the metabolites produced. Ma et al. reported that reducing the abundance of intestinal Clostridial bacteria can increase PBAs levels and inhibit liver tumors. Moreover, DCA can increase TLR2 expression in HSCs together with increased TLR2 agonist lipoteichoic acid, which results in the tumor-promoting SASP (Loo et al., 2017).

The onset and development of primary liver cancer are modulated by SBAs through several different mechanisms, including DNA injury, inflammation-related tumorigenesis, and hepatotoxicity (Yoshimoto et al., 2013; Bourzac, 2014), which favor an immunosuppressive tumor microenvironment by reducing the accumulation of natural killer T (NKT) cells in the liver (Hartmann and Kronenberg., 2018; Ma et al., 2018; Mossanen et al., 2019) Another mechanism by which intestinal microorganism-induced changes in the metabolism of bile acids control the growth of liver cancer is *via* the regulation of CXC chemokine ligand 16 (CXCL16) expression in the liver and the recruitment of NKT cells by CXCL16 (Ma et al., 2018). PBAs have been shown to play a key role in the up-regulation of CXC16 in endothelial cells in the liver sinus, which in turn contributes to the recruitment of NKT cells. NKT cells subsequently kill tumor cells in a CD1-dependent manner. In another study, antibiotics inhibited the development of liver cancer induced by high cholesterol and a fat NASH diet, along with a significant reduction in SBAs, which activate the mammalian target of rapamycin (mTOR) pathway in liver cells (Yamada et al., 2018).

The liver influences the Th17 and Treg balance of the intrinsic layer *via* a derivative of lithocholic acid (LCA), a bile acid metabolite. 3-OxoLCA can combine with retinoic acid-associated orphan receptors γT (ROR γT) to prevent the proliferation of Th17 cells, while isoalloLCA produces mitochondrial ROS, leading to the up-regulation of forkhead box protein 3 (FOXP3), a regulator of Treg differentiation. In mice fed a Lieber DeCarli ethanol diet, the farnesoid X receptor (FXR), a regulator of bile acid and lipid metabolism, was lowered, resulting in an increase in bile acid levels produced and secreted by the liver. Degeneration of liver fat and ALD are likely to occur in mice depleted by FXR, as well as in chronic feeding models with excess ethanol. Experimental treatment of DK-naive T cells isolated from mice defected by FXR with isoalloLCA and 3-oxolca did not alter the expression of FOXP3 in relation to the control group. Moreover, in cells treated with 3-oxoLCA, FXR did not help inhibit Th17 cells (Bruellman and Llorente., 2021).

### SCFAs

SCFAs represent an important category of bacterial metabolites, and are considered to be the richest microbiome-derived metabolites in the gut (Zhang Z. et al., 2019). Intestinal flora produce various gastrointestinal enzymes, including propionate and acetate coenzyme A transferase, and butyrate kinase, all of which transfer complex or undigested carbohydrates of diets into host absorbable SCFAs, mainly acetic acid, propionic acid, and butyrate (El Kaoutari et al., 2013; Wisniewski et al., 2019). For example, Bacteroidetes produce acetate and propionate, which can be delivered directly through the portal vein to peripheral tissues, including the liver and adipose tissue, for lipogenesis and gluconeogenesis (Koh et al., 2016; Wilson et al., 2017). In addition, SCFAs have a strong ability to inhibit intestinal inflammation and prevent pathogen invasion, as well as to maintain barrier integrity, primarily by activating GPCRs or inducing their inhibitory effects on HDACs to further influence gene expression (Zhang Z. et al., 2019). Although SCFAs are generally considered health-promoting factors, particularly for colonic epithelial cells, one study showed that high dietary consumption of inulin, a non-absorbable fiber that is converted to butyrate, promoted the development of HCC in mice with dysbacteriosis (Singh et al., 2018). Evidence has shown that an imbalance between T helper 17 cells (Th17s) and regulatory T cells (Tregs) is associated with aberrant immune responses. Recent advances in culture-independent techniques for the detection and identification of intestinal commensal bacteria enabled the discovery that Th17 and Treg differentiation are regulated by SCFAs, particularly butyrate, produced by the gut microbiota. This finding provided a mechanistic link between dysbiosis, defined as changes in the composition of the gut microbiota, and various inflammatory diseases. On this basis, research suggested that dysbiosis with reduced production of SCFAs leading to Th17/Treg imbalance, is involved in the etiology of liver cancer. Thus, the inhibition of fermentation by drugs or the depletion of fermentation bacteria can significantly reduce SCFAs and prevent HCC (Singh et al., 2018). In contrast, another study showed that propionate improves the cytotoxic effect of cisplatin on liver cancer by modulating the G-protein coupled receptor 41 (GPR41) signaling pathway (Kobayashi et al., 2018).

### Other Gut Metabolites

**FIGURE 2:**
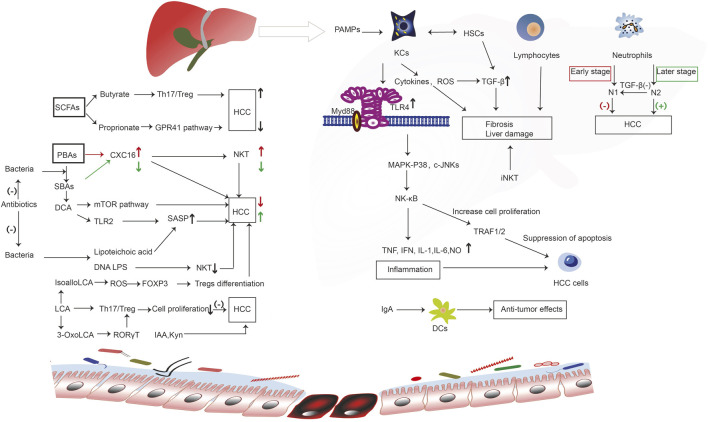
The immune molecular mechanism of intestinal microorganisms and their metabolites in the occurrence and development of liver cancer. Bacterial components such as LPS and SCFA butyrate bind to receptors to regulate the activity of immune cells. Liver-derived PBAs enter the intestinal tract and generate SBAs under the action of bacteria, which promotes the occurrence of liver cancer through a series of immune reactions. However, SCFA proprionate binds to GPR41 receptor, and the microbial metabolism produces IAA and Kyn to inhibit the occurrence of liver cancer through a series of reactions. Enterogenous microorganism and metabolic products reach the liver, activating liver immune cells, including KCs, lymphocytes, and neutrophils; these cell surface receptors and PAMPs undergo ligand binding, triggering a series of immune cascade, induce inflammation, promote or inhibit liver cell hyperplasia of fibers, liver cell proliferation, differentiation, and apoptosis, thus playing a role in promoting or inhibiting liver cancer; Intestinal DCs can interact with IgA that is released by the liver to impart an anti-cancer effect. However, SCFAs and BAs (PBAs, SBAs, DCA, LCA) produced by intestinal microorganisms can bind to corresponding receptors, trigger a series of immune responses, regulate CXC16 levels, SASP, NKT, Treg differentiation, and Th17/Treg, and promote or inhibit liver cancer. CXC16, CXC chemokine ligand 16; c-JNKs, c-Jun N-terminal kinases; DCs, dendritic cells; DCA, deoxycholic acid; FOXP3, forkhead box protein 3; GPR41, G-protein coupled receptor 41; HCC, hepatocellular carcinoma; HSCs, hepatic stellate cells; iNKT, invariant natural killer; IAA, indole-3-acetic acid; IFN, interferon; IL-1, interleukin-1; IL-6, interleukin-6; KCs, kupffer cells; Kyn, kynurenine; LCA, lithocholic acid; LTA, lipoteichoic acid; MAPK, mitogen-activated protein kinase; mTOR, mammalian target of rapamycin; NF-kB, nuclear factor-kB; N1, neutrophils 1; N2, neutrophils 2; NKT, natural killer; NO, nitric oxide; PBAs, primary bile acids; RORγT, retinoic acid-associated orphan receptors γT ; ROS, reactive oxygen species; SCFAs, short-chain fatty acids; SASP, senescence-associated secretory phenotype; SBAs, secondary bile acids; TLR, toll-like receptor; TGF-β1, transforming growth factor-β1; Th17/Treg, T helper 17/ regulatory T; TRAF1/2, tumor necrosis receptor associated factors 1/2.

Indol-3-acetic acid (IAA), an intestinal-derived metabolite, reduces high-fat diet-induced hepatotoxicity by improving liver lipogenesis, lipid metabolism, and inflammatory and oxidative stress in mice (Ji et al., 2019). Moreover, in SK-HEP-1-implanted nude mice, IAA can induce tumor regression, and may represent a novel cancer therapeutic agent (Park et al., 2009). Another study showed the IAA confers protection against HCC by affecting antioxidant gene expression and DNA fragmentation (Mourão et al., 2009). As an endogenous ligand of the human activation of the aryl hydrocarbon receptor, the tryptophan (Trp) catabolite kynurenine (Kyn) is a liver-derived Trp-degrading enzyme that may be associated with HCC (Opitz et al., 2011). The inability of D-Trp-6-luteinizing hormone releasing hormone cannot inhibit the development of HCC (Guéchot et al., 1989). The transcription of enzymes implicated in L-Trp metabolism causes DNA injury during the early stages of tumorigenesis (Tummala et al., 2014). (Figure 2).

In short, gut microbiota and their metabolites reach the liver and activate liver immune cells, including KCs, lymphocytes, and neutrophils, inhibiting or promoting the occurrence and development of liver cancer through various immune response mechanisms. Intestinal DCs interact with IgA released by the liver, thereby producing anti-cancer effects. Intestine microbe metabolites (e.g., PBAs, SBAs, DCA, LCA, SCFAs) regulate the development and progression of liver cancer by stimulating or inhibiting immune reactions and inflammation in the liver.

In addition, we also focused on the relationship between the changes of liver cancer biomarkers and intestinal microflora and metabolites. As it is well known, serum alpha-fetoprotein (α-AFP), structure-specific recognition protein 1 and lamin B1 are important biomarkers for the diagnosis of liver cancer, as well as important indicators for the evaluation of therapeutic effect and prognosis (Feng et al., 2021). Studies have shown that these biomarkers are involved in the immune and inflammatory responses in the development and progression of liver cancer, among which, the level of α-AFP is associated with the diversity of intestinal microorganisms (Zhang et al., 2019a). Elshaer et al. reported that in thioacetame-induced liver cirrhosis rats, the TLR4-CXCL9 pathway was activated and the serum α-AFP level increased, which could be prevented by administration of *Lactobacillus* Plantarum (Elshaer et al., 2019). Another study reported that the levels of serum zonulin, a marker of intestinal permeability, increased significantly in patients with liver cirrhosis and HCC. The combination of zonulin and AFP exhibited a significantly larger receiver operating characteristic curve compared to zonulin or AFP alone, suggesting that their combination confers significant benefit to diagnostic accuracy in differentiating liver cirrhosis from HCC (Wang et al., 2019). However, to the best of our knowledge, there are limited studies on the effects of intestinal bacteria and metabolites on liver cancer biomarkers. In future, it will be possible to pay attention to their relationship, or even combine their detection, to provide a basis for the diagnosis, treatment, and prognosis of liver cancer.

## Potentiating the Anticancer Effects of Therapeutic Drugs by Regulating the Gut Microbiota

Changes in intestinal flora are associated with resistance to chemotherapy drugs. Using antibiotics, post-biotics, probiotics, fecal microflora transplant (FMT), or nanotechnologies, to regulate microflora may enhance the anti-cancer effects of chemotherapy agents (Compare et al., 2017; Cheng et al., 2020).

### Antibiotics

Studies have shown that antibiotic treatment at advanced stages of HCC in mice may be effective in reducing cancer development (Meroni et al., 2019). For example, rifaximine is currently being tested on the microflora in various clinical trials of CLD as the safest non-absorbable antibiotic (Wiest et al., 2017). Rifaximine is a broad-spectrum compound that reduces endotoxin and anti-inflammatory effects independent of its bactericidal effects. The combination of rifaximine and simvastatin for the treatment of key mechanisms of liver cirrhosis progression, namely liver and intestinal and systemic inflammatory reactions, is currently undergoing a clinical phase 3, multicenter, double-blind, placebo-controlled trial to prevent ACLF in patients with decompensated cirrhosis. Recent studies have shown that the levels of some SBAs in the liver decreased after antibiotics depleted commensal bacteria from the gut microbiome in mice. This relieved inhibition of CXCL16, a potent recruiter of NKT cells, and also increased the levels of certain PBAs known to induce CXCL16, resulting in the accumulation of hepatic NKT cells and a reduction in liver tumors.

### Postbiotics

Recent data indicate that the potential mechanisms for controlling micro-organism-based intestinal homeostasis depend on their metabolites, also known as postbiotics (Tsilingiri and Resscigno., 2013). Postbiotics are advanced microbiology tools that can be used to maintain long-term health benefits. Their compounds are variable and depend on the strain and their metabolic state and include SCFAs, SBAs, proteins, enzymes, peptides, bacteriocins, polysaccharides, vitamins and organic acids (Compare et al., 2017), all of which have been described as having an immuno-regulatory and protective effect in the intestinal barrier. Postbiotics can strengthen the structure of the close bonding of the epithelium by increasing the expression of TJs proteins and intestinal mucin, which can promote the restoration of intestinal barrier function (Tsilingiri et al., 2012). In particular, postbiotic agents can act on immune cells and protect intestinal tissues from immunopathological damage by increasing the secretion of anti-inflammatory cytokines such as IL-10 (Mileti et al., 2009; Compare et al., 2017).

Postbiotic agents also manifest as factors inducing the elasticity of the microflora. They may act as inhibitors of pathogenic bacteria or possibly as signal quorum molecules, regulating the density of bacterial cells and supporting the formation of biological membranes of microbial composition (Fanning et al., 2012). Studies have shown that postbiotic agents have anti-proliferation, anti-inflammatory, and anti-cancer properties, and are able to regulate the effectiveness of cancer treatments and reduce the side effects of traditional treatments on patients with cancer (Rad et al., 2021).

### Probiotics

Numerous clinical trials have been conducted to study the effects of prebiotics/probiotics on cancer. Some trials showed improved clinical outcomes in patients using probiotics (Mego et al., 2015; Theodoropoulos et al., 2016; Flesch et al., 2017; Tian et al., 2019), while others were unable to verify the significant effects of receiving probiotics. A prospective clinical study in patients with colorectal cancer showed that *Lactobacillus acidophilus* NCFM and *Bifidobacterium lactis* Bl-04 increased the levels of *butyrate-producing bacteria,* including *Faecalibacterium and Clostridiales* spp.*,* but decreased the level of bacteria associated with colorectal cancer, such as *Fusobacterium* and *Peptostreptococcus* (Hibberd et al., 2017). In addition to altering the features of microorganisms, probiotics have also been reported to suppress the development of cancer in animal models. Zhang *et al.* established a model of liver cancer in rats using diethylnitroamine to reveal that oral VSL#3 probiotic mixture reduced intestinal inflammatory reactions, maintained the integrity of the intestinal mucosa, and inhibited tumor growth (Zhang et al., 2012). A subsequent study showed that the probiotic mixture Prohep decreased the number of Th17 cells in the tumor and thus inhibited the development of liver cancer in a mouse model grafted under the skin (Li et al., 2016). Clinical trials evaluating the therapeutic potential of VSL#3 in patients with liver cirrhosis (Dhiman et al., 2014) or NAFLD (Anderson et al., 2004) have shown that probiotics alleviate the severity of diseases closely related to the progression of liver cancer (Michelotti et al., 2013).

However, some clinical trials have found no evidence of the clinical benefits of probiotics in the treatment of cancer (McNaught et al., 2002; Anderson et al., 2004). Postoperative treatment of patients with head and neck cancer with mixed strains of *Lactobacillus* and Bifidobacterium did not improve clinical outcomes. Moreover, patients treated with probiotics and those treated with placebo were shown to have similar postoperative infection rates, inflammatory markers, and levels of diaminoxydase, which are indicators of intestinal permeability (Lages et al., 2018). The conflicting results of clinical trials can be explained by differences in the microbiome and host genome between individuals. Colonization and function of probiotics are affected by native microflora, host gene expression features, and other exogenous factors (Morgan et al., 2012; Lages et al., 2018). Evaluating the clinical benefits of probiotics in patients with cancer therefore remains a challenge. Moreover, most probiotic clinical trials have limitations such as reduced sample size, short duration of treatment, and lack of follow-up of long-term effects of probiotics on patients. Thus, well-designed studies are essential to evaluate the probiotic treatment of cancer patients with cancer.

### FMT

FMT is defined as the digestive transmission of intestinal microflora from healthy donors to unhealthy recipients, with the objective of restoring intestinal homeostasis or establishing a new balance to eliminate or improve disease (van Nood et al., 2013). FMT has been recognized by official guidelines as the standard treatment for recurrent *clostridium difficile* infection (CDI), with a cure rate close to 90% (van Nood et al., 2013; Chen et al., 2019). A previous clinical showed that patients with liver cirrhosis and recurrent liver encephalopathy were well tolerated and safe in the long term by oral administration in capsules after pre-treatment with antibiotics (Bajaj et al., 2018; Bajaj et al., 2019a; Bajaj et al., 2019b). Moreover, FMT has been shown to restore antibiotic-related microbiological biodiversity and reduce function destruction, resulting in continuous improvement in cognitive function parameters and reduced rates of liver encephalopathy re-emergence and liver-related hospitalization (Bajaj et al., 2018; Bajaj et al., 2019a; Bajaj et al., 2019b). FMT has also been shown to reverse early portal hypertension, intra-hepatic endothelial dysfunction, and insulin resistance in rats on a high-fat, fructose diet (Garcia-Lezana et al., 2018). Generally, to make FMT more viable in the treatment of cancer, the choice of the ideal donor remains a crucial question, as preliminary evidence indicates that the donor’s intestinal microbiome is a determining factor in the response rate to the patient in cancerous mice (Routy et al., 2018). However, until we can identify a microflora that supports cancer immunotherapy, therapists should make use of the balanced fecal microflora of healthy donors rather than the patient’s disturbed microflora. There is no consensus on which species or combinations of bacteria are the best option to enhance immune effects, and further research is needed in this area.

### Nanoparticles

**FIGURE 3:**
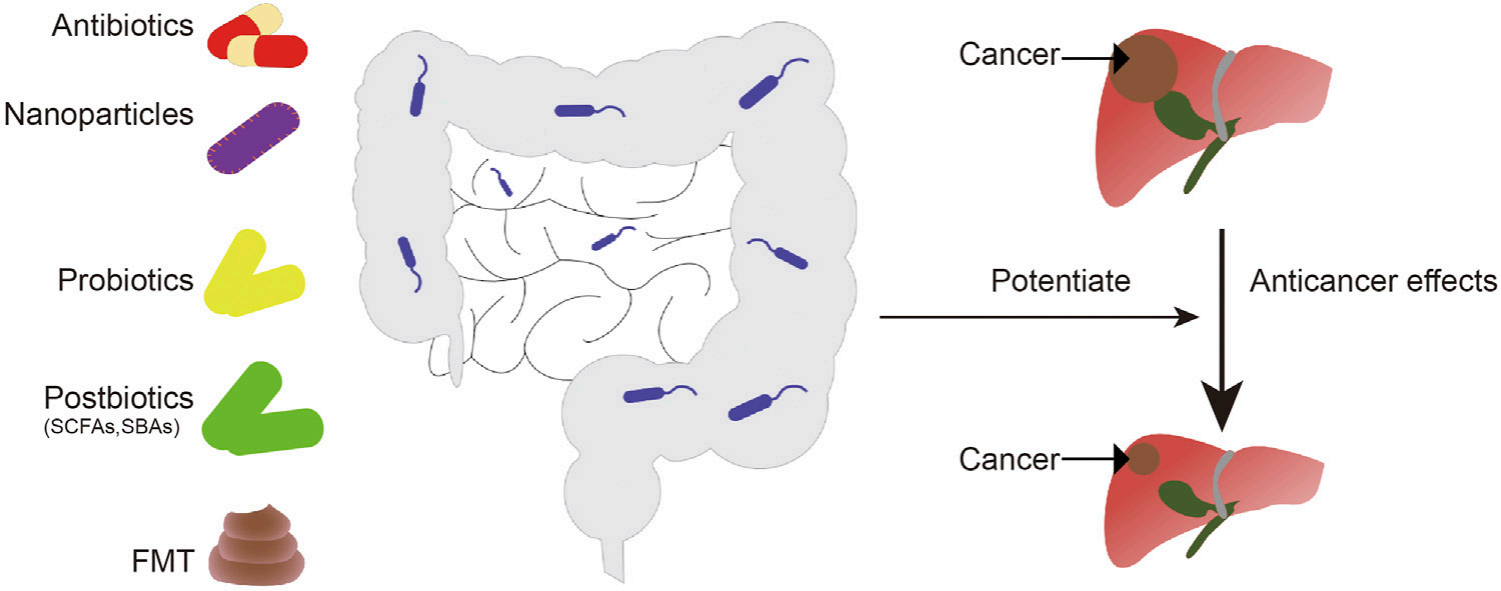
Intestinal microorganisms can be regulated by antibiotics, probiotics, postbiotics, fecal bacteria transplantation, and nanoparticles to enhance the anti-liver cancer effect of chemotherapy drugs. FMT, fecal microbiota transplantation; SCFAs, short-chain fatty acids; SBAs, secondary bile acids.

Ongoing clinical trials have shown that nanotechnology can be used to target cancer-related bacteria or to release anticancer agents in a controlled manner, with fewer side reactions (Angsantikul et al., 2018; Song et al., 2019). Given the impact of nanotechnology on cancer prevention and treatment, the assessment of toxicity, side effects, and downstream mechanisms mediated by nanoparticles should be considered. In addition, the interactions between NPs and the immune system may have effects on the gut microbiota. For example, exosome-like NP (ELNs) RNAs regulate gut microbiota to enhance the function of the gut barrier (Teng et al., 2018), and phage-guided irinotecan-loaded dextran NPs promote release of bacterial derived butyrate, which may improve the therapeutic strategy of tumor (Kannen et al., 2019). By combining Fe@Fe3O4 NPs with ginsenoside Rg3 (NpRg3), a new nano-drug is currently being developed, with good efficacy for liver cancer (Ren et al., 2020). (Figure 3).

## Conclusion and Perspectives

In conclusion, this review summarizes the molecular immune mechanisms of gut microbiota in the occurrence and development of liver cancer, revealing the important role of the microbiota-gut-liver axis on liver cancer. In addition, we describe how to balance the intestinal flora by regulating diet, antibiotics, prebiotics, postbiotics, and fecal bacteria transplantation to improve the treatment of liver cancer.

However, this paper still has some limitations. In this review, there are more studies on the cell and animal models, but fewer clinical studies, especially on the relationship between microbial metabolites and liver cancer, which need to be further strengthened. In terms of the molecular immune mechanism, there are still many problems that need to be resolved in the study of microbes and metabolites and liver cancer; for example, whether SCFAs play a role in the occurrence and development of liver cancer and whether there are differences between different types and different individuals, particularly individual differences in microbes and whether the results are consistent in animals and humans. Second, further investigations on the regulation of intestinal microbiota to enhance the efficacy of tumor chemotherapy and preclinical and clinical studies on HCC are warranted. In conclusion, intestinal microbes and their metabolites are closely related to the occurrence and development of liver cancer. Regulating intestinal microorganisms represents a promising strategy to enhance the efficacy of immunotherapy in liver cancer and reduce adverse reactions.
